# Integrated physical education course experience and exercise adherence tendency among undergraduates in Sichuan Province, China: chain mediation and configurational evidence from SEM and fsQCA

**DOI:** 10.3389/fpsyg.2026.1902785

**Published:** 2026-08-18

**Authors:** Jun Xia, Yu-Xuan Yang

**Affiliations:** Sports Institute, Chengdu University of Technology, Chengdu, Sichuan, China

**Keywords:** chain mediation, exercise adherence tendency, exercise psychological need satisfaction, fsQCA, health belief, integrated physical education course experience, undergraduates in Sichuan Province

## Abstract

**Objective:**

This study examined the association between integrated physical education course experience and exercise adherence tendency among undergraduates in Sichuan Province, China. It focused on indirect associations involving exercise psychological need satisfaction and health belief and on configurations associated with high exercise adherence tendency.

**Methods:**

A cross-sectional survey was conducted among 3,391 undergraduates from 25 universities in Sichuan Province, China. Structural equation modeling (SEM) was used to examine a theory-informed chain mediation model linking integrated physical education course experience, exercise psychological need satisfaction, health belief, and exercise adherence tendency. Bootstrap analysis with 5,000 resamples assessed direct and indirect associations, and fuzzy-set qualitative comparative analysis (fsQCA) identified sufficient configurations associated with high exercise adherence tendency.

**Results:**

Integrated physical education course experience showed a positive direct association with exercise adherence tendency (*β* = 0.131, 95% CI [0.061, 0.198]). The specific indirect associations through exercise psychological need satisfaction (*β* = 0.110), health belief (*β* = 0.185), and the sequential pathway through exercise psychological need satisfaction and health belief (*β* = 0.209) were significant. The sequential pathway accounted for the largest specific indirect proportion of the total association (32.86%), and the total indirect association accounted for 79.40%. The fsQCA identified six partially overlapping sufficient configurations (solution coverage = 0.516; solution consistency = 0.891). Health belief appeared in all six configurations, and exercise psychological need satisfaction appeared in five.

**Conclusion:**

Exercise adherence tendency among undergraduates in Sichuan Province was associated with the interplay of course experience, psychological need satisfaction, and health belief. The SEM and fsQCA findings provide complementary variable-oriented and configurational evidence. Given the cross-sectional design, the results should be interpreted as associations rather than causal effects.

## 1 Introduction

Physical inactivity remains a major public health challenge. Regular physical activity supports physical and mental health and helps prevent noncommunicable diseases (WHO, 2020). A pooled analysis of 507 surveys found that 31.3% of adults worldwide were insufficiently active in 2022 (Strain et al., 2024). University students warrant particular attention because routines and health-related beliefs are still developing, and their activity is shaped by environmental, social, motivational, emotional, and capability-related factors (Brown et al., 2024). Understanding the conditions associated with sustained exercise participation during this period is therefore an educational and public health priority.

University physical education (PE) offers a structured setting in which students encounter instruction, repeated practice, assessment, peer interaction, and embodied learning. Quality PE is expected to develop competence, motivation, confidence, knowledge, and positive orientations toward continued physical activity (UNESCO, 2015). In Chinese higher education, reforms increasingly connect sport, education, and health promotion (Zhang and Yang, 2025). Students’ evaluations of PE involve teaching characteristics, teacher characteristics and attitudes, course experience, learning atmosphere, facilities, and examination (Jin and Xi, 2026), indicating that course experience is a multidimensional educational construct.

Research has often examined PE satisfaction, physical activity, exercise motivation, or health belief separately. Course satisfaction alone, however, does not explain why students report a stronger tendency to maintain exercise. Positive course experiences may become relevant to sustained participation when they are associated with internalized motivation and personally meaningful health-related cognition (Biscardi et al., 2022; Yan et al., 2025). A central gap is therefore to clarify the psychological and cognitive pathways linking integrated PE course experience with current exercise adherence tendency.

Self-determination theory provides the motivational component of this explanation. Satisfaction of the needs for autonomy, competence, and relatedness is associated with more self-endorsed engagement and sustained behavior (Ryan and Deci, 2000). Evidence from exercise research supports the relevance of autonomous motivation and need-related processes (Teixeira et al., 2012), and the Chinese version of the Basic Psychological Needs in Exercise Scale has been validated among undergraduates (Liu et al., 2013). A supportive PE course may therefore be associated with exercise adherence tendency through students’ exercise psychological need satisfaction.

The Health Belief Model provides a complementary cognitive component. Health-related behavior is associated with awareness of health issues, perceived susceptibility and severity, perceived benefits and barriers, and self-efficacy (Rosenstock, 1974; Rosenstock et al., 1988). In PE, students may connect exercise with health value, the risks of inactivity, and confidence in maintaining activity. Research in Chinese youth and young adults has linked sports-related health communication and health belief with sport behavior (Sun et al., 2024). Examining psychological need satisfaction and health belief together can therefore show how motivational experience and health-related evaluation are jointly associated with exercise adherence tendency.

Variable-oriented analysis alone may not capture the multiple combinations through which high exercise adherence tendency occurs. SEM can estimate average theory-informed associations and indirect pathways, whereas fsQCA can identify conjunctural conditions, equifinality, and asymmetric set relations (Ragin, 2008; Fiss, 2011). Combining the two approaches allows the average structural pattern to be considered alongside alternative sufficient configurations.

Accordingly, this study examines the association between integrated PE course experience and exercise adherence tendency among undergraduates in Sichuan Province. It evaluates indirect associations through exercise psychological need satisfaction and health belief, provides a stronger theoretical account of the proposed motivational-to-cognitive ordering, and identifies configurations associated with high exercise adherence tendency. The study extends PE course research beyond satisfaction, integrates self-determination theory with the health belief perspective, and demonstrates the complementary value of SEM and fsQCA without treating cross-sectional associations as evidence of temporal causality.

## 2 Literature review and hypotheses development

### 2.1 Integrated physical education course experience and exercise adherence tendency

Integrated Physical Education Course Experience (IPECE) refers to students’ overall perceptions of the instructional, interpersonal, experiential, environmental, and evaluative features of university PE courses. In Chinese higher education, it includes teaching characteristics, teacher characteristics and attitudes, course experience, learning atmosphere, facilities, and examination. These dimensions jointly represent students’ PE course satisfaction and course-related experience (Jin and Xi, 2026).

Exercise adherence tendency (EA) refers to a relatively stable, self-reported inclination to maintain regular, persistent, and effortful exercise. It is reflected in behavioral habits, effort commitment, and emotional experience. Studies of Chinese college students have linked exercise adherence with social support, subjective exercise experience, commitment, self-efficacy, and emotional regulation (Tian and Shi, 2022; Wang et al., 2026). In the present study, EA denotes current adherence tendency rather than objectively verified lifelong behavior.

University PE courses provide repeated opportunities for exercise within an organized educational setting. Students who perceive teaching, teacher support, course activities, the learning atmosphere, facilities, and assessment more positively may also report more stable routines, greater effort commitment, and more positive exercise-related experiences. IPECE is therefore expected to show a positive association with EA, while the cross-sectional design does not establish temporal direction.

*H1*. Integrated physical education course experience is positively associated with exercise adherence tendency among undergraduates.

### 2.2 Exercise psychological need satisfaction as a motivational pathway

Exercise Psychological Need Satisfaction (EPNS) refers to the satisfaction of autonomy, competence, and relatedness in exercise contexts. Autonomy reflects self-endorsed participation, competence reflects perceived capability, and relatedness reflects support and connection with others. Self-determination theory identifies these needs as central to internalized motivation and sustained engagement (Ryan and Deci, 2000). The Basic Psychological Needs in Exercise Scale measures the three dimensions (Vlachopoulos and Michailidou, 2006), and its Chinese version has shown satisfactory validity and reliability among undergraduates (Liu et al., 2013).

PE course experience is relevant to EPNS because students encounter teachers, peers, tasks, assessment systems, and physical environments during participation. Choices can support autonomy, progressive tasks and feedback can support competence, and inclusive teacher-peer interaction can support relatedness. Need-supportive processes have been associated with engagement, effort, persistence, and adaptive outcomes in PE and exercise settings (Standage et al., 2005; Teixeira et al., 2012).

Within the proposed framework, EPNS represents a motivational pathway linking IPECE with EA. Students who report supportive, meaningful, and competence-enhancing course experiences may be more likely to regard exercise as self-endorsed and to maintain it as part of their routine. The proposed mediation is interpreted as an indirect association rather than a causal process.

*H2*. Exercise psychological need satisfaction accounts for an indirect association between integrated physical education course experience and exercise adherence tendency.

### 2.3 Health belief as a cognitive pathway

Health Belief (HB) refers here to health awareness, perceived susceptibility to health risks, perceived severity of health risks, and exercise-related self-efficacy. The Health Belief Model proposes that health behavior is associated with risk appraisal, expected consequences, perceived benefits and barriers, and confidence in taking action (Rosenstock, 1974; Rosenstock et al., 1988). Exercise research has used health-related beliefs to explain why individuals value physical activity, evaluate the risks of inactivity, and feel capable of engaging in health-promoting behavior (Glanz et al., 2008; Faghih et al., 2024; Sahan, 2024).

In Chinese youth and young adult samples, Sun et al. (2024) operationalized health belief through health awareness, perceived susceptibility, perceived severity, and self-efficacy when examining sport behavior. Research among Chinese college students has also linked physical health beliefs with exercise behavioral intention (Zhang et al., 2024). These findings support the relevance of health-related cognition in university students’ exercise decisions.

University PE can function as a structured health education environment. Through course content, teacher guidance, assessment, and embodied participation, students may become more attentive to health, more aware of the consequences of inactivity, and more confident in exercising. HB therefore represents a cognitive pathway through which IPECE may be indirectly associated with EA (Faghih et al., 2024).

*H3*. Health belief accounts for an indirect association between integrated physical education course experience and exercise adherence tendency.

### 2.4 Chain mediation of exercise psychological need satisfaction and health belief

EPNS and HB represent distinct but connected components of the proposed framework. IPECE is an external educational context, EPNS captures students’ proximal motivational experience in exercise, and HB captures their cognitive evaluation of health value, risk, and capability. When students feel autonomous, competent, and socially supported, they may be more receptive to health information, more likely to regard exercise as personally relevant, and more confident in managing exercise-related health behavior. EPNS may therefore provide a motivational foundation for stronger health belief (Huang C. et al., 2025).

This ordering is consistent with self-determination theory, which links need-supportive contexts with internalization and self-endorsed regulation (Ryan and Deci, 2000), and with the Health Belief Model’s emphasis on self-efficacy in translating health concerns into action (Rosenstock et al., 1988). Experiences of autonomy and competence may strengthen confidence in exercise, while relatedness may increase openness to teacher and peer health messages. These experiences can be associated with recognizing the health significance of activity and the risks of inactivity (Warburton and Bredin, 2017). The motivational and cognitive components may therefore be sequentially related rather than functioning only in parallel (Zhou et al., 2025).

The proposed order was specified on theoretical grounds. It should be interpreted as a theory-informed structural ordering, not as an empirically verified temporal sequence. The model therefore examines whether IPECE is associated with EPNS, whether EPNS is associated with HB, and whether HB is associated with EA within the same cross-sectional covariance structure.

*H4*. Exercise psychological need satisfaction and health belief sequentially account for an indirect association between integrated physical education course experience and exercise adherence tendency.

### 2.5 Configurational perspective based on fsQCA

The hypotheses can be examined with SEM, which estimates net structural associations and specific indirect pathways. High EA, however, may be associated with different combinations of course, motivational, and cognitive conditions. One student may report high EA in the presence of strong psychological need satisfaction and health belief, whereas another may do so when comprehensive course conditions and health belief occur together. Such patterns involve configurational complexity, conjunction, equifinality, and asymmetry.

fsQCA complements net-association methods by examining whether combinations of conditions are sufficient for an outcome (Ragin, 2008). It also distinguishes core, peripheral, absent, and irrelevant conditions by comparing solution types (Fiss, 2011). Recent work has shown that high physical activity among Chinese university students can be associated with multiple configurations rather than a single necessary condition (Jia et al., 2025).

**Figure 1:**
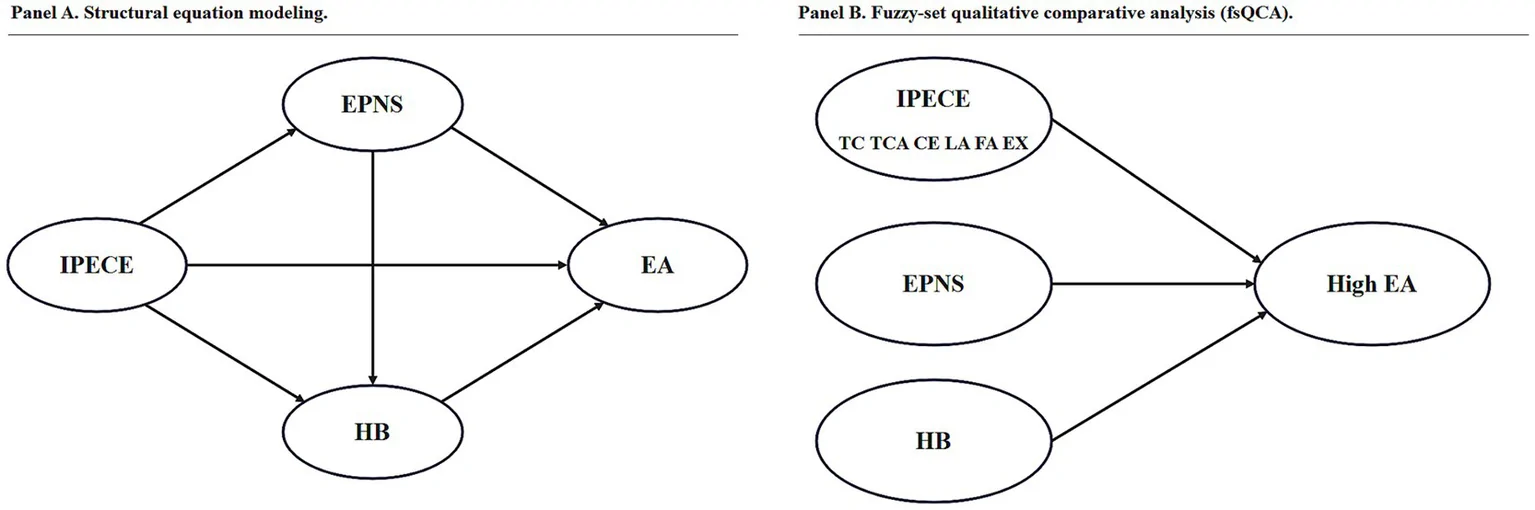
Theoretical model of SEM pathways and fsQCA configurational logic. **(A)** Presents the SEM framework, including the direct association between integrated physical education course experience and exercise adherence tendency, the separate indirect pathways through exercise psychological need satisfaction and health belief, and the sequential indirect pathway through exercise psychological need satisfaction and health belief. **(B)** Presents the fsQCA framework, in which the dimensions of integrated physical education course experience, exercise psychological need satisfaction, and health belief are treated as configurational conditions associated with high exercise adherence tendency. IPECE, Integrated physical education course experience; EPNS, Exercise psychological need satisfaction; HB, Health belief; EA, Exercise adherence tendency; TC, Teaching characteristics; TCA, Teacher characteristics and attitudes; CE, Course experience; LA, Learning atmosphere; FA, Facilities; EX, Examination.

SEM and fsQCA were therefore used for different but complementary purposes. SEM examined the direct association and the separate and sequential indirect associations among IPECE, EPNS, HB, and EA. fsQCA identified alternative combinations of the six IPECE dimensions, EPNS, and HB associated with high EA. The theoretical model is presented in Figure 1.

## 3 Method

### 3.1 Participants and procedure

The study protocol was reviewed and approved by the Ethics Committee of the Sports Institute, Chengdu University of Technology, China. The study was conducted in accordance with the ethical principles of voluntary participation, informed consent, anonymity, and confidential data use. No personally identifiable information was collected, and the data were used only for academic research purposes.

Data were collected between March 10, 2026 and April 10, 2026. Participants were recruited from 25 universities in Sichuan Province, China, including Chengdu University of Information Technology, Sichuan University, University of Electronic Science and Technology of China, Southwestern University of Finance and Economics, Southwest Jiaotong University, Southwest Petroleum University, Chengdu Technological University, and other regular undergraduate institutions. The survey was administered online through Wenjuanxing, a widely used Chinese online survey platform. The survey link was distributed through student affairs offices, physical education instructors, class WeChat groups, and university course platforms. Participation was voluntary, anonymous, and unrelated to students’ course grades or academic evaluation. Participants were informed of the research purpose, estimated completion time, confidentiality procedures, and their right to withdraw before submitting the questionnaire. Electronic written informed consent was obtained from all participants before they proceeded to the substantive survey items.

The study protocol was reviewed and approved by the Ethics Committee of the Sports Institute, Chengdu University of Technology, China. The ethics approval number was CDUT2026TY006. The study was conducted in accordance with the ethical principles of voluntary participation, informed consent, anonymity, and confidential data use. No personally identifiable information was collected, and the data were used only for academic research purposes. To reduce potential common method bias, the questionnaire introduction emphasized that there were no right or wrong answers, that responses would be analyzed only in aggregate form, and that participants should answer honestly according to their own experiences (Podsakoff et al., 2003).

A total of 3,612 questionnaires were initially collected. Since all questionnaire items were set as mandatory in Wenjuanxing, there were no missing values in the raw dataset. Following recommendations for detecting careless responses in online survey data, a multi-criterion screening procedure was applied rather than relying on a single exclusion rule (Meade and Craig, 2012). Questionnaires were excluded if they met any of the following criteria: (a) the respondent did not meet the eligibility criteria, such as being younger than 18 years old, not being a full-time undergraduate student, or having no recent college physical education course experience; (b) the completion time was less than 100 s, which was below one-third of the median completion time and was therefore considered too short for attentive completion of the questionnaire; (c) the respondent failed the attention-check item embedded in the questionnaire, for example, “To confirm that you are reading the items carefully, please select ‘Agree’ for this item”; (d) the questionnaire showed highly patterned responding, such as choosing the same response option for nearly all substantive items; or (e) duplicate submissions were identified based on the platform’s available technical indicators, such as IP address and device identifier. After excluding invalid responses, 3,391 valid questionnaires remained, representing 93.88% of the initially collected questionnaires.

The final sample size was sufficient for the planned analyses. SEM requirements depend on model complexity, number of indicators, factor loadings, misspecification, and expected association sizes rather than on a single universal rule (Wolf et al., 2013). Large samples are generally desirable for models involving multiple latent constructs and indirect pathways (Kline, 2016). The final sample of 3,391 participants provided adequate power for the measurement and structural analyses. The fsQCA component included eight configurational conditions, producing 2^8^ = 256 logically possible truth-table rows; the sample provided broad empirical coverage for examining configurations associated with high and non-high exercise adherence tendency. Participant characteristics are shown in Table 1.

**Table 1:** Demographic and background characteristics of the participants (*N* = 3,391).

Characteristic	Category	n	%
Gender	Male	1,718	50.66
	Female	1,654	48.78
	Other / prefer not to say	19	0.56
Age	18 years	641	18.90
	19 years	829	24.45
	20 years	807	23.80
	21 years	689	20.32
	22 years or older	425	12.53
	M ± SD	19.91	1.43
Grade	First year	846	24.95
	Second year	915	26.98
	Third year	861	25.39
	Fourth year	769	22.68
Academic discipline	Humanities and social sciences	683	20.14
	Science and engineering	1,364	40.22
	Economics and management	594	17.52
	Medicine/health-related disciplines	309	9.11
	Arts	238	7.02
	Other disciplines	203	5.99
University type	Comprehensive university	1,048	30.91
	Science and engineering university	882	26.01
	Finance/economics university	421	12.42
	Normal university	358	10.56
	Applied undergraduate college	682	20.11
PE course experience	Currently taking a college PE course	2,365	69.74
	Completed a college PE course within the most recent semester	1,026	30.26
Weekly exercise frequency	0–1 time per week	528	15.57
	2 times per week	862	25.42
	3 times per week	1,053	31.05
	4 times per week or more	948	27.96
Participation in sport organizations	No sport club or university team participation	2,354	69.42
	Sport club participation	827	24.39
	University team participation	210	6.19

### 3.2 Measurements

All constructs were measured using previously validated Chinese or China-context instruments. Items were assessed on a five-point Likert scale from 1 = strongly disagree to 5 = strongly agree, with higher scores indicating higher levels of the corresponding construct. Confirmatory factor analyses were conducted to examine the item-level first-order structure, the four second-order constructs, construct reliability, convergent validity, and discriminant validity before the structural model was evaluated.

Integrated Physical Education Course Experience (IPECE) was measured using the Physical Education Course Satisfaction Scale for Chinese College Students (PECSS-CCS) developed by Jin and Xi (2026). The scale was originally developed for Chinese college students and contains 26 items across six first-order dimensions: teaching characteristics (TC; 3 items), teacher characteristics and attitudes (TCA; 6 items), course experience (CE; 4 items), learning atmosphere (LA; 4 items), facilities (FA; 5 items), and examination (EX; 4 items). The original study reported satisfactory reliability and validity for the six-factor structure of the PECSS-CCS and confirmed its applicability to Chinese college students (Jin and Xi, 2026). In the present study, these six dimensions were specified as first-order factors loading onto the second-order construct of IPECE. A representative item is: “The course helps me improve my sport skills.” Higher scores indicated a more positive and supportive physical education course experience.

Exercise Psychological Need Satisfaction (EPNS) was measured using the Basic Psychological Needs in Exercise Scale (BPNES). The BPNES was originally developed to assess the satisfaction of three basic psychological needs in exercise settings—autonomy, competence, and relatedness—and has been validated in Chinese undergraduate samples (Liu et al., 2013; Vlachopoulos and Michailidou, 2006). The Chinese validation study provided evidence of factorial validity, internal reliability, measurement invariance across sex, and nomological validity among Hong Kong undergraduate students (Liu et al., 2013). The scale contains 12 items across three first-order dimensions: autonomy need satisfaction (AUT; 4 items), competence need satisfaction (COM; 4 items), and relatedness need satisfaction (REL; 4 items). In the present study, these three dimensions were modeled as first-order factors loading onto the second-order construct of EPNS. A representative item is: “The way I exercise is consistent with my own choices and interests.” Higher scores indicated that students experienced greater autonomy, competence, and relatedness during exercise and physical education participation.

Health Belief (HB) was measured using the health belief scale adopted in Sun et al.’s (2024) study on sports social media health communication, adolescent health beliefs, and sport behavior. This instrument is suitable for the present study because it was used in a Chinese youth and young adult context, with participants aged 14–24 years, and was analyzed using PLS-SEM (Sun et al., 2024). Following the scale structure reported in that study, HB was measured through four first-order dimensions: health awareness (HA; 6 items), perceived susceptibility to health risks (PS; 3 items), perceived severity of health risks (DS; 3 items), and self-efficacy (SE; 4 items). In the present study, these four dimensions were specified as first-order factors loading onto the second-order construct of HB. A representative item is: “I pay attention to health risks caused by insufficient physical activity.” Higher scores indicated stronger health awareness, higher perceived health-risk susceptibility and severity, and greater confidence in performing health-promoting exercise behaviors.

Exercise adherence tendency (EA) was measured using the Exercise Adherence Scale (EAS) developed by Wang et al. (2016) and subsequently used in Chinese college student samples. EA refers in this study to students’ current self-reported tendency to maintain regular, persistent, and effortful exercise participation. The EAS contains 14 items across behavioral habits (BH; 4 items), effort commitment (EC; 6 items), and emotional experience (EE; 4 items). Recent studies have reported satisfactory reliability and model fit for the scale (Chen et al., 2024; Wang et al., 2026). BH, EC, and EE were specified as first-order factors loading on the second-order construct of EA. A representative item is: “I can keep participating in physical exercise regularly.” Higher scores indicated stronger exercise adherence tendency and a stronger orientation toward sustained physical activity; they did not constitute evidence of objectively verified lifelong behavior.

### 3.3 Analytical strategy

Data were analyzed using SPSS 29.0, AMOS 26.0, and fsQCA 3.0. SPSS was used for descriptive statistics, reliability analysis, correlation analysis, common-method diagnostics, and data preparation. AMOS was used for item-level and dimension-level confirmatory factor analyses, SEM, bootstrap tests, and supplementary covariate-adjusted models. fsQCA was used for calibration, necessity and sufficiency analyses, solution comparison, and configurational robustness checks.

Potential common method bias was assessed because all focal variables were collected through the same self-reported questionnaire. Harman’s single-factor test and a single-factor CFA were used as diagnostic procedures (Podsakoff et al., 2003; Fuller et al., 2016). These procedures can indicate whether a dominant common factor is evident, but they cannot fully exclude bias arising from same-source measurement.

Measurement assessment proceeded at both item and dimension levels. An item-level hierarchical CFA specified all 68 items as indicators of 16 correlated first-order factors, which in turn loaded on IPECE, EPNS, HB, and EA. A correlated 16-factor model was also estimated for comparison. Standardized factor loadings, Cronbach’s alpha, composite reliability (CR), and average variance extracted (AVE) were examined. Values of 0.70 or above for alpha and CR and 0.50 or above for AVE were treated as acceptable (Nunnally and Bernstein, 1994; Fornell and Larcker, 1981; Hair et al., 2019). Detailed item-level results are provided in Supplementary Tables S1–S4.

Discriminant validity was evaluated using the Fornell-Larcker criterion and the heterotrait-monotrait ratio of correlations (HTMT). Discriminant validity is supported under the Fornell-Larcker criterion when the square root of AVE for each construct exceeds its correlations with other constructs (Fornell and Larcker, 1981). HTMT values below 0.85 were treated as evidence of acceptable discriminant validity (Henseler et al., 2015).

The primary SEM examined the theory-informed chain model among IPECE, EPNS, HB, and EA. Mean scores for the first-order dimensions were used as manifest indicators of the corresponding second-order constructs. This dimension-level specification matched the higher-order conceptual structure and reduced model complexity. Model fit was evaluated using χ^2^/df, CFI, TLI, SRMR, and RMSEA, with conventional criteria applied (Hu and Bentler, 1999; Kline, 2016). Because the primary SEM used dimension-level indicators, its global fit indices describe how closely the model reproduced the dimension-level covariance matrix. They should not be interpreted as evidence of error-free item-level measurement or as confirmation of temporal or causal direction.

Structural associations were summarized using standardized path coefficients, *p* values, and R^2^ for endogenous constructs. A bootstrap procedure with 5,000 resamples was used to estimate direct, specific indirect, total indirect, and total associations. An indirect association was considered statistically significant when its 95% bootstrap confidence interval did not include zero (Preacher and Hayes, 2008).

Supplementary robustness analyses adjusted the focal structural model for gender, age, grade, academic discipline, university type, PE-course status, and sport-organization participation. Age was entered as a continuous covariate. The categorical variables were represented by k − 1 dummy variables, with male, first-year students, humanities and social sciences, comprehensive universities, current PE-course participation, and no sport-organization participation as the reference categories. All covariates were entered simultaneously as predictors of EPNS, HB, and EA, and covariances among the covariates were freely estimated. Weekly exercise frequency was represented by three dummy variables only in the sensitivity model, with 0–1 time per week as the reference category, because of its conceptual proximity to exercise adherence tendency. Robustness was evaluated by comparing the direction, statistical significance, and magnitude of the six focal structural paths and the R^2^ values across models.

fsQCA was conducted to identify configurations of course experience, psychological need satisfaction, and health belief associated with high exercise adherence tendency. It was used to examine configurational complexity, conjunctural relations, equifinality, and asymmetry (Ragin, 2008; Schneider and Wagemann, 2012). The conditions were TC, TCA, CE, LA, FA, EX, EPNS, and HB, and high EA was the outcome.

Raw scores were transformed into fuzzy-set membership scores through direct calibration. In the absence of external qualitative thresholds for the Likert-type measures, the 95th, 50th, and 5th percentiles served as empirical anchors for full membership, the crossover point, and full non-membership, respectively, following established fsQCA practice (Ragin, 2008; Fiss, 2011).

Necessity analysis examined the presence and absence of each condition, with consistency of 0.90 used as the threshold for necessity (Schneider and Wagemann, 2012). For sufficiency analysis, the truth table was minimized using the Quine-McCluskey algorithm. The case-frequency threshold was 10, raw consistency of 0.80 and PRI consistency of 0.70 were used as retention criteria, and complex, parsimonious, and intermediate solutions were generated (Ragin, 2008; Greckhamer et al., 2018). Core and peripheral conditions were identified by comparing the parsimonious and intermediate solutions: conditions present in both were classified as core, whereas conditions present only in the intermediate solution were classified as peripheral.

Configurational robustness was assessed by changing the calibration anchors to 4, 3, and 2 and by varying the case-frequency threshold from 10 to 5 and 15. Stability was evaluated by comparing the principal configurations and the recurrence of key conditions across specifications (Schneider and Wagemann, 2012; Greckhamer et al., 2018).

## 4 Results

### 4.1 Common method bias

Procedural measures were used to reduce potential common method bias. The questionnaire was anonymous, participants were told that there were no right or wrong answers, responses were used only for academic research and analyzed in aggregate, and the instructions encouraged answers based on personal experience.

Harman’s single-factor test extracted 16 factors with eigenvalues greater than 1, accounting for 67.130% of the total variance. The first factor explained 27.813%, below the commonly used 40% diagnostic threshold. A single-factor CFA also fit the data poorly: χ^2^ = 55,302.499, df = 2,210, χ^2^/df = 25.024, CFI = 0.534, TLI = 0.520, SRMR = 0.080, and RMSEA = 0.084, 90% CI = [0.084, 0.085]. These results did not indicate a dominant single-method factor. Harman’s test and the single-factor CFA are diagnostic procedures, however, and cannot fully rule out common method bias in same-source self-report data.

### 4.2 Descriptive statistics, reliability, and validity

An item-level hierarchical CFA was conducted using all 68 items. The model specified 16 first-order factors loading on four second-order constructs and showed excellent fit: χ^2^(2,188) = 2,540.886, CFI = 0.997, TLI = 0.997, SRMR = 0.015, and RMSEA = 0.007, 90% CI [0.006, 0.008]. The correlated 16-factor model also fit well, χ^2^(2,090) = 2,445.374, CFI = 0.997, TLI = 0.997, SRMR = 0.013, and RMSEA = 0.007. The second-order model did not fit significantly worse, Δχ^2^(98) = 95.512, *p* = 0.552, and had lower AIC and BIC values. Standardized item loadings ranged from 0.583 to 0.875 and were all significant at *p* < 0.001. Across the 16 first-order dimensions, Cronbach’s *α* ranged from 0.797 to 0.902, CR ranged from 0.803 to 0.902, and AVE ranged from 0.505 to 0.665. Detailed results are presented in Supplementary Tables S1–S4.

Descriptive statistics, internal consistency reliability, composite reliability, and convergent validity for the dimension-level SEM constructs are reported in Table 2. The standardized loading ranges in this table were estimated from the dimension-level model, in which the first-order dimension means served as indicators of the four higher-order constructs; the item-level hierarchical CFA estimates are reported separately in Supplementary Table S4. Mean scores ranged from 3.426 to 3.597. Cronbach’s α values ranged from 0.879 to 0.931, CR values ranged from 0.798 to 0.889, standardized dimension loadings ranged from 0.721 to 0.774, and AVE values ranged from 0.558 to 0.591. These results supported the reliability and convergent validity of the four higher-order constructs.

**Table 2:** Descriptive statistics, reliability, and convergent validity in the dimension-level measurement model.

Variable	Items	M	SD	Cronbach’s α	Std. dimension loading	CR	AVE
IPECE	26	3.597	0.673	0.931	0.747–0.762	0.889	0.571
EPNS	12	3.426	0.753	0.879	0.737–0.771	0.798	0.568
HB	16	3.435	0.724	0.910	0.721–0.772	0.835	0.558
EA	14	3.475	0.788	0.911	0.765–0.774	0.813	0.591

Estimates in this table were obtained from the dimension-level measurement/structural model, in which first-order dimension means served as indicators of the four higher-order constructs. These values should be distinguished from the item-level hierarchical CFA estimates reported in Supplementary Table S4. M, mean; SD, standard deviation; α, Cronbach’s alpha; CR, composite reliability; AVE, average variance extracted; Std. dimension loading, standardized dimension-level factor-loading range. IPECE, integrated physical education course experience; EPNS, exercise psychological need satisfaction; HB, health belief; EA, exercise adherence tendency.

Discriminant validity was evaluated using both the Fornell–Larcker criterion and the heterotrait–monotrait ratio of correlations. As shown in Table 3, the square root of AVE for each construct exceeded its correlations with the other constructs, supporting discriminant validity.

**Table 3:** Correlations and discriminant validity of the SEM constructs.

Variable	IPECE	EPNS	HB	EA
IPECE	0.756			
EPNS	0.594^***^	0.754		
HB	0.564^***^	0.552^***^	0.747	
EA	0.510^***^	0.491^***^	0.589^***^	0.769

Values on the diagonal in bold are the square roots of AVE. Discriminant validity is supported when the square root of AVE for each construct exceeds its correlations with the other constructs. IPECE, integrated physical education course experience; EPNS, exercise psychological need satisfaction; HB, health belief; EA, exercise adherence tendency. ***p < 0.001.

The heterotrait–monotrait ratio of correlations (HTMT) was also used to further assess discriminant validity. As shown in Table 4, all HTMT values ranged from 0.637 to 0.762, which were well below the conservative threshold of 0.85. These results provided additional evidence that the four constructs were empirically distinct.

**Table 4:** HTMT ratios of the SEM constructs.

Variable	IPECE	EPNS	HB	EA
IPECE	—			
EPNS	0.758	—		
HB	0.694	0.732	—	
EA	0.637	0.661	0.762	—

HTMT, heterotrait-monotrait ratio of correlations. Values below 0.85 indicate acceptable discriminant validity. IPECE, integrated physical education course experience; EPNS, exercise psychological need satisfaction; HB, health belief; EA, exercise adherence tendency.

### 4.3 Structural equation modeling results

The proposed dimension-level SEM showed excellent fit to the data: χ^2^ = 92.763, df = 98, χ^2^/df = 0.947, *p* = 0.630, CFI = 1.000, TLI = 1.000, SRMR = 0.009, and RMSEA = 0.000, 90% CI [0.000, 0.008]. The model was estimated with maximum likelihood and no correlated residuals were added. The exceptionally favorable indices indicate close reproduction of the covariance matrix among the dimension-level indicators. They do not imply an absence of item-level measurement error, validate the proposed temporal ordering, or establish causal direction.

**Figure 2:**
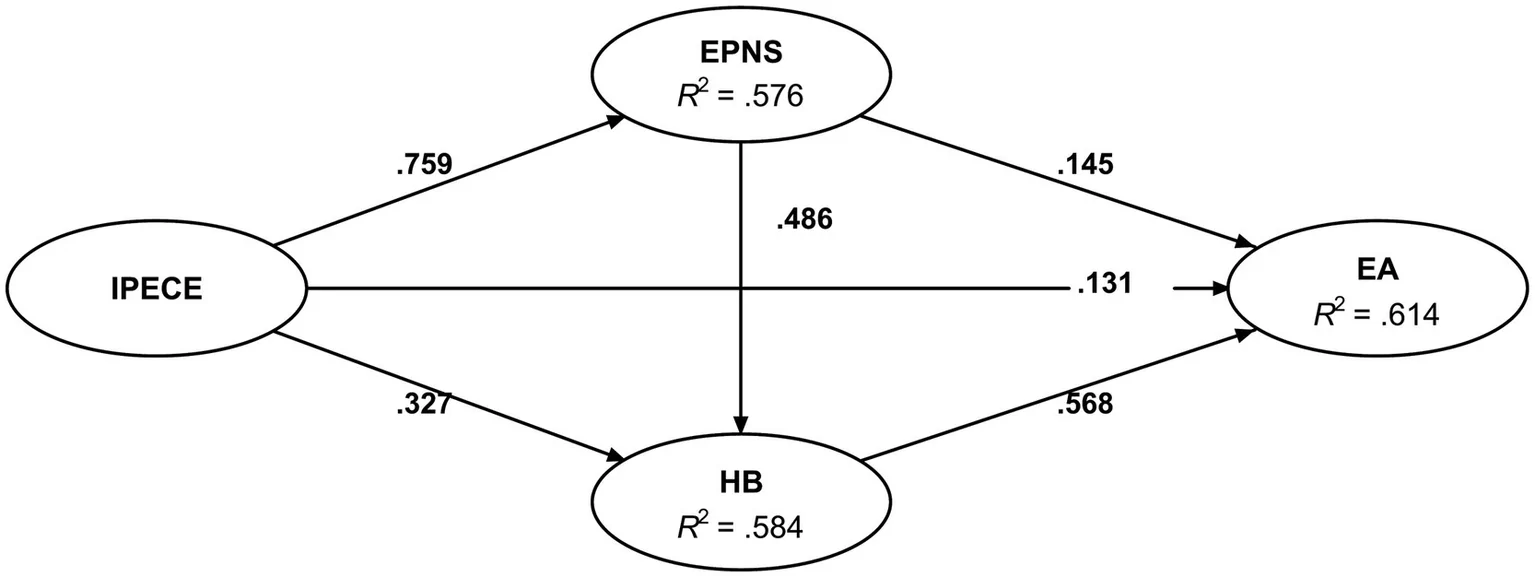
Standardized structural associations and explained variance in the dimension-level SEM. Only the six standardized structural coefficients and *R*^2^ values are displayed. Standardized dimension loadings estimated in the dimension-level model are reported in Table 2, whereas loadings from the 68-item hierarchical CFA are reported in Supplementary Table S4. IPECE, integrated physical education course experience; EPNS, exercise psychological need satisfaction; HB, health belief; EA, exercise adherence tendency. Solid arrows represent the theory-informed structural paths.

Figure 2 presents the six standardized structural path coefficients and the explained variance. IPECE explained 57.6% of the variance in EPNS. IPECE and EPNS jointly explained 58.4% of the variance in HB, and IPECE, EPNS, and HB jointly explained 61.4% of the variance in EA.

All six hypothesized structural associations were statistically significant (Table 5). IPECE showed a positive direct association with EA (*β* = 0.131, *p* < 0.001), supporting H1. IPECE was positively associated with EPNS (β = 0.759, p < 0.001) and HB (*β* = 0.327, *p* < 0.001). EPNS was positively associated with EA (*β* = 0.145, *p* < 0.001) and HB (β = 0.486, *p* < 0.001). HB showed the strongest standardized association with EA among the direct paths (*β* = 0.568, *p* < 0.001).

**Table 5:** Standardized structural associations in the dimension-level SEM.

Path	Standardized β	*p*
IPECE → EA	0.131	< 0.001
IPECE → EPNS	0.759	< 0.001
EPNS → EA	0.145	< 0.001
IPECE → HB	0.327	< 0.001
HB → EA	0.568	< 0.001
EPNS → HB	0.486	< 0.001

Standardized β values are reported for interpretation, and p values correspond to the significance tests of the estimated structural paths. IPECE, integrated physical education course experience; EPNS, exercise psychological need satisfaction; HB, health belief; EA, exercise adherence tendency.

Bootstrap estimates with 5,000 resamples are shown in Table 6. The direct association between IPECE and EA was significant (β = 0.131, 95% CI [0.061, 0.198]) and accounted for 20.60% of the total association. The specific indirect association through EPNS was significant (*β* = 0.110, 95% CI [0.052, 0.173]), supporting H2, as was the indirect association through HB (*β* = 0.185, 95% CI [0.143, 0.231]), supporting H3. The sequential indirect association through EPNS and HB was also significant (β = 0.209, 95% CI [0.173, 0.253]), supporting H4. It accounted for 32.86% of the total association and was the largest specific indirect component. The total indirect association was 0.505, representing 79.40% of the total association of 0.636.

**Table 6:** Bootstrap analysis of direct and indirect associations.

Association type	Path	Hypothesis	β	Boot SE	Boot LLCI	Boot ULCI	Ratio %
Direct	IPECE → EA	H1	0.131	0.035	0.061	0.198	20.60
Specific indirect	IPECE → EPNS → EA	H2	0.110	0.031	0.052	0.173	17.30
Specific indirect	IPECE → HB → EA	H3	0.185	0.022	0.143	0.231	29.09
Sequential indirect	IPECE → EPNS → HB → EA	H4	0.209	0.021	0.173	0.253	32.86
Total indirect	IPECE → Mediators → EA	—	0.505	0.028	0.453	0.563	79.40
Total	IPECE → EA	—	0.636	0.015	0.607	0.665	100

β, standardized association; Boot SE, bootstrap standard error; Boot LLCI, lower limit of the 95% bootstrap confidence interval; Boot ULCI, upper limit of the 95% bootstrap confidence interval. Bootstrap sample size = 5,000. An indirect association is statistically significant when the 95% bootstrap confidence interval excludes zero. IPECE, integrated physical education course experience; EPNS, exercise psychological need satisfaction; HB, health belief; EA, exercise adherence tendency.

Supplementary robustness analyses adjusted for gender, age, grade, academic discipline, university type, PE-course status, and sport-organization participation. All six focal structural associations retained the same direction and remained significant at p < 0.001; the largest absolute change in the reported standardized coefficients was 0.005. The R^2^ for EA changed from 0.614 to 0.618. A sensitivity model that additionally adjusted for weekly exercise frequency produced the same substantive pattern. The coding scheme, reference categories, and covariate path specification are reported in Supplementary Table S5.

### 4.4 Fuzzy-set qualitative comparative analysis results

To complement the SEM results and examine configurational complexity, fsQCA was conducted with high exercise adherence tendency as the outcome. The configurational model was specified as EA = f(TC, TCA, CE, LA, FA, EX, EPNS, HB).

Raw scores were calibrated into fuzzy-set membership scores using the empirical anchors shown in Table 7. The 95th, 50th, and 5th percentiles represented full membership, the crossover point, and full non-membership, respectively.

**Table 7:** Calibration anchors for fsQCA condition and outcome sets.

Set	Full membership	Crossover point	Full non-membership
TC	5.000	3.667	2.000
TCA	4.667	3.667	1.833
CE	4.750	3.750	1.750
LA	4.750	3.750	2.500
FA	5.000	3.800	2.000
EX	5.000	3.750	2.250
EPNS	4.583	3.417	2.167
HB	4.521	3.479	2.250
EA	4.639	3.528	2.167

Calibration anchors transformed raw scores into fuzzy-set membership scores. In the absence of external qualitative thresholds, the 95th, 50th, and 5th percentiles were used as empirical anchors for full membership, the crossover point, and full non-membership, respectively (Ragin, 2008; Fiss, 2011). TC, teaching characteristics; TCA, teacher characteristics and attitudes; CE, course experience; LA, learning atmosphere; FA, facilities; EX, examination; EPNS, exercise psychological need satisfaction; HB, health belief; EA, exercise adherence tendency.

No single condition reached the conventional necessity threshold of 0.90 (Table 8). Consistency values for the presence conditions ranged from 0.709 to 0.776; HB had the highest consistency for high EA (0.776), followed by EPNS (0.758) and EX (0.739). The absence conditions also remained below 0.90. High exercise adherence tendency therefore was not contingent on any single condition alone.

**Table 8:** Necessary condition analysis for high exercise adherence tendency.

Condition	High EA consistency	High EA coverage	Non-high EA consistency	Non-high EA coverage
TC	0.724	0.734	0.541	0.535
~TC	0.540	0.547	0.730	0.721
TCA	0.731	0.720	0.543	0.522
~TCA	0.514	0.536	0.709	0.720
CE	0.709	0.727	0.528	0.529
~CE	0.541	0.540	0.727	0.709
LA	0.713	0.729	0.526	0.524
~LA	0.534	0.536	0.728	0.712
FA	0.725	0.729	0.536	0.525
~FA	0.527	0.538	0.723	0.720
EX	0.739	0.721	0.550	0.523
~EX	0.511	0.538	0.706	0.725
EPNS	0.758	0.745	0.532	0.510
~EPNS	0.502	0.524	0.734	0.747
HB	0.776	0.780	0.500	0.490
~HB	0.492	0.502	0.775	0.772

“~” denotes the absence or low level of a condition. A condition is generally considered necessary when consistency is at least 0.90. TC, teaching characteristics; TCA, teacher characteristics and attitudes; CE, course experience; LA, learning atmosphere; FA, facilities; EX, examination; EPNS, exercise psychological need satisfaction; HB, health belief; EA, exercise adherence tendency.

The truth table was minimized using the Quine-McCluskey algorithm with a case-frequency threshold of 10. Rows meeting the specified consistency and PRI criteria were retained. The complex, parsimonious, and intermediate solutions were identical. The use of logical remainders therefore did not yield additional simplification, and every retained condition in the reported configurations was classified as a core condition.

Six partially overlapping sufficient configurations associated with high membership in exercise adherence tendency were identified (Table 9). Solution coverage was 0.516 and solution consistency was 0.891. Raw coverage ranged from 0.378 to 0.404, consistency ranged from 0.911 to 0.921, and unique coverage ranged from 0.020 to 0.030. The low unique coverage values indicate substantial overlap among the configurations; they should therefore be interpreted as related sufficient patterns rather than mutually exclusive pathways.

**Table 9:** Configurational solutions associated with high exercise adherence tendency.

Conditions	Type I		Type II	Type III		
	S1	S2	S3	S4	S5	S6
TC	●	●	●	●	●	
TCA			●	●	●	●
CE	●	●	●	●		●
LA	●		●	●	●	●
FA		●	●	●	●	●
EX	●	●	●		●	●
EPNS	●	●		●	●	●
HB	●	●	●	●	●	●
Raw coverage	0.398	0.404	0.379	0.378	0.382	0.380
Unique coverage	0.024	0.030	0.021	0.020	0.025	0.022
Consistency	0.914	0.911	0.918	0.921	0.921	0.920
Solution coverage	0.516					
Solution consistency	0.891					

● = core presence; blank = irrel evant or “do not care.” Core and peripheral conditions were determined by comparing the parsimonious and intermediate solutions. Because the complex, parsimonious, and intermediate solutions were identical, all retained conditions were core presences; no peripheral or absent conditions occurred in the six configurations. The case-frequency threshold was 10. The relatively low unique coverage values indicate that the configurations partially overlap. Type I, teaching-course experience-assessment with psychological need and health belief configuration; Type II, comprehensive course experience with health belief configuration; Type III, teacher/environment support with psychological need and health belief configuration. TC, teaching characteristics; TCA, teacher characteristics and attitudes; CE, course experience; LA, learning atmosphere; FA, facilities; EX, examination; EPNS, exercise psychological need satisfaction; HB, health belief; EA, exercise adherence tendency.

Type I comprised S1 and S2. Both configurations contained the core presence of TC, CE, EX, EPNS, and HB. S1 additionally contained core LA, whereas S2 additionally contained core FA. The pair indicates two closely related patterns in which instructional, experiential, evaluative, motivational, and cognitive conditions occur together, with learning atmosphere or facilities differentiating the configurations.

Type II comprised S3. It contained the core presence of all six IPECE dimensions together with HB, while EPNS was irrelevant to this configuration. This does not indicate that EPNS was generally unimportant. It indicates that, within S3, high EA was sufficiently associated with comprehensive positive course conditions and HB without EPNS serving as a defining condition.

Type III comprised S4, S5, and S6. These configurations shared the core presence of TCA, LA, FA, EPNS, and HB. EX was irrelevant in S4, CE was irrelevant in S5, and TC was irrelevant in S6. The shared pattern highlights the recurrent conjunction of teacher support, the learning environment, facilities, psychological need satisfaction, and health belief, while the relevance of particular course-related conditions varied.

HB appeared as a core presence in all six configurations, and EPNS appeared as a core presence in five. Their recurrence is consistent with their importance in the SEM, but it does not establish necessity: neither condition reached the 0.90 necessity threshold. The configuration metrics likewise indicate overlap rather than six fully independent pathways.

Robustness checks changed the calibration anchors to 4, 3, and 2 and varied the case-frequency threshold from 10 to 5 and 15. The principal configurational patterns remained stable, as did the recurrence of HB and the frequent presence of EPNS. The findings were therefore not materially altered by these reasonable specification changes.

## 5 Discussion

### 5.1 Interpretation of the SEM indirect associations

This study examined the association between integrated PE course experience and exercise adherence tendency among undergraduates in Sichuan Province through exercise psychological need satisfaction and health belief. The results place university PE within a broader motivational and cognitive context. Because all variables were measured concurrently, the proposed chain should be interpreted as a theory-informed structural pattern rather than a verified temporal process.

IPECE showed a positive direct association with EA, but the direct component represented only 20.60% of the total association. The three indirect components together represented 79.40%, and the sequential EPNS-HB component was the largest. Students reporting more positive PE course experiences also tended to report stronger psychological need satisfaction and health belief, which were in turn associated with stronger EA. This pattern is consistent with the view that educational experiences become behaviorally relevant when they are connected with motivation, confidence, perceived value, and self-regulation (Ng et al., 2012; Standage et al., 2005).

The strong association between IPECE and EPNS supports the relevance of self-determination theory in university PE. Teaching, teacher attitudes, course experience, learning atmosphere, facilities, and examination jointly shape whether students experience choice, progress, competence, and interpersonal support. Need-supportive PE environments have been associated with adaptive motivation, engagement, and positive experiences (Vasconcellos et al., 2020), and recent work continues to emphasize competence, autonomous motivation, and supportive interpersonal contexts in physical activity participation (Ntoumanis and Moller, 2025). The present results extend this reasoning to multidimensional PE course experience among undergraduates in Sichuan Province.

HB showed the strongest direct association with EA and accounted for a larger specific indirect component than EPNS alone. Enjoyment or competence during class may be insufficient for sustained participation unless students also recognize the health value of exercise, understand the risks of inactivity, and feel capable of maintaining activity. This interpretation is consistent with evidence linking exercise-related health belief with physical activity among Chinese college students (Gong and Sheng, 2022; Sheng et al., 2023).

The sequential association offers a theoretically coherent link between the two frameworks. IPECE represents the educational context, EPNS represents a proximal motivational experience, and HB represents health-related cognitive evaluation. Students who feel autonomous, competent, and supported may be more receptive to health information and more confident that exercise is valuable and manageable. Health belief may then connect this motivational basis with adherence tendency (Ryan et al., 2009; Ren and Song, 2025). The ordering is theoretically plausible, but longitudinal evidence is required to determine whether changes occur in this sequence over time.

The stronger HB-EA association also suggests that health belief may be more proximal to students’ evaluation of why exercise should continue beyond course requirements. EPNS may support self-endorsed and meaningful participation, whereas HB may help students connect exercise with personal health goals and perceived capability. Affective and motivational experiences remain relevant to future activity, but they may not fully explain adherence without health-related interpretation and confidence (Rhodes and Kates, 2015; Huang D. et al., 2025).

### 5.2 Configurational interpretation of high exercise adherence tendency

The fsQCA findings complement the average structural associations by showing that high EA was linked to multiple conjunctural patterns. The six configurations were partially overlapping rather than mutually exclusive, as indicated by their low unique coverage. This pattern is consistent with equifinality: students may report high EA under different combinations of course, motivational, and cognitive conditions.

No single condition was necessary for high EA. HB had the highest necessity consistency, followed by EPNS, but both remained below 0.90. Their relevance therefore lies in how they combine with other conditions. HB was recurrent across all six sufficient configurations, while EPNS occurred in five, yet high EA did not depend on either condition in isolation.

Type I combined the core presence of teaching characteristics, course experience, examination, EPNS, and HB with either learning atmosphere or facilities. The two configurations share a common instructional, experiential, evaluative, motivational, and cognitive base. Their difference indicates that a positive learning atmosphere and adequate facilities can characterize alternative contextual variants within an otherwise similar pattern.

Type II combined all six course-experience dimensions with HB, while EPNS was irrelevant to that configuration. The pattern qualifies a purely linear reading of the SEM. EPNS was important in the average structural model and in most configurations, but high EA was also sufficiently associated with comprehensive course conditions and HB when EPNS was not a defining element of S3.

Type III combined teacher characteristics and attitudes, learning atmosphere, facilities, EPNS, and HB, while TC, CE, or EX was irrelevant in one of the three configurations. Teacher and environmental support therefore appeared repeatedly with motivational and cognitive conditions, although the relevance of specific course features varied across configurations.

All retained conditions were classified as core because the complex, parsimonious, and intermediate solutions were identical. This result means that logical remainders did not further simplify the observed patterns under the specified analysis. It does not make each configuration independent or imply that every recurrent condition is necessary. The distinction between recurrence, sufficiency, and necessity is central to configurational interpretation (Greckhamer et al., 2018).

### 5.3 Integration of SEM and fsQCA findings

The two methods converged on the importance of motivational and cognitive conditions. In the SEM, HB had the strongest direct association with EA, and the sequential EPNS-HB pathway was the largest specific indirect component. In the fsQCA, HB was present in all six configurations and EPNS in five. This convergence strengthens the interpretation that health-related cognition and psychological need satisfaction are consistently relevant across both average-association and set-theoretic analyses.

The methods also supplied different information. SEM summarized the average associations among four higher-order constructs and quantified the relative contribution of indirect pathways. fsQCA showed that high EA could be associated with several conjunctions of lower-level course conditions, including a configuration in which EPNS was irrelevant and variants in which learning atmosphere, facilities, teaching characteristics, course experience, or examination differed in relevance. The configurational evidence therefore adds equifinality, conjunction, and partial substitutability to the net-association account rather than duplicating it.

### 5.4 Theoretical implications

The study extends PE course research by treating course experience as a multidimensional educational context linked to exercise adherence tendency, rather than limiting the analysis to satisfaction as an endpoint. The findings connect teaching, teacher attitudes, course experience, learning atmosphere, facilities, and examination with motivational and cognitive processes relevant to sustained exercise orientation.

The integration of self-determination theory and the Health Belief Model places psychological need satisfaction within a broader educational and health-related framework. Need-supportive experiences were associated with HB as well as EA, suggesting a motivational-to-cognitive structural sequence. Health belief was also embedded in the PE context rather than treated solely as an individual disposition. Teacher communication, assessment, peer atmosphere, and experiences of autonomy and competence may all be related to how students interpret exercise as personally relevant and manageable (Vasconcellos et al., 2020; Gong and Sheng, 2022; Zhang and Miao, 2025). This interpretation is compatible with behavior-change perspectives that emphasize capability, opportunity, and motivation (Michie et al., 2011).

The hybrid design contributes methodologically by separating two questions. SEM assessed whether the hypothesized constructs followed the expected average structural pattern, whereas fsQCA identified combinations sufficient for high EA. The convergence on HB and EPNS, together with the configurational variation in course conditions, shows why net associations and set-theoretic sufficiency can provide complementary evidence.

The findings also relate to physical literacy and continued physical activity. Physical literacy frameworks connect participation with competence, motivation, confidence, knowledge, and health-related understanding (Cairney et al., 2019). The present data concern current adherence tendency, not lifelong behavior, but they suggest how university PE may contribute to an orientation toward sustained physical activity when positive course experiences are associated with motivational need satisfaction and health-related meaning.

### 5.5 Practical implications

The findings suggest that university PE may benefit from being designed as an integrated learning environment. Teaching quality, teacher attitudes, course activities, learning atmosphere, facilities, and examination were jointly relevant in the structural and configurational analyses. Attention to only one element may therefore be insufficient. Course planning may be better aligned with sustained exercise participation when pedagogical, interpersonal, environmental, evaluative, motivational, and health-related components are considered together (Xu et al., 2025).

Pedagogical practices can support autonomy, competence, and relatedness. Meaningful choices in activity, intensity, practice, and personal goals can support autonomy. Progressive tasks, differentiated instruction, feedback, and assessment of improvement can support competence. Cooperative learning, peer encouragement, inclusive climates, and respectful teacher-student interaction can support relatedness (Melguizo-Ibáñez et al., 2025). Such practices may be especially useful for students with low confidence or negative prior PE experiences (Guo et al., 2025).

Health belief education can be embedded in practical activity rather than delivered only as abstract information or fear-based warnings (Çiftci and Kadıoğlu, 2023). Teachers can help students connect intensity, recovery, posture, endurance, strength, and emotional regulation with concrete health outcomes. Progress monitoring and realistic exercise planning may also help students interpret activity as personally meaningful and manageable beyond the course.

Assessment warrants similar attention because examination appeared in several configurations. PE assessment may benefit from reducing reliance on one-time performance tests (Uraipong et al., 2025) and incorporating attendance, effort, personal improvement, exercise planning, skill progression, application of health knowledge, and reflective records (Nolan et al., 2026). Such practices may support sustained engagement and self-regulation rather than privileging prior athletic ability.

The three configurational types offer differentiated guidance. Type I emphasizes the conjunction of teaching, course experience, assessment, EPNS, and HB with learning atmosphere or facilities. Type II emphasizes comprehensive course quality together with HB. Type III emphasizes teacher and environmental support together with EPNS and HB, while particular course features vary. These patterns can help institutions prioritize combinations of modifiable conditions rather than assuming that one universally dominant intervention is sufficient (Jia et al., 2025).

PE reform may also be coordinated with campus health promotion. Courses provide a structured context, while accessible exercise spaces, peer communities, sport clubs, and campus norms can support participation outside class (Ndupu et al., 2023). Coordinated action among PE departments, student affairs offices, sport organizations, and health education units may better address capability, opportunity, and motivation together (Michie et al., 2011).

### 5.6 Limitations and future research

The cross-sectional design prevents conclusions about temporal ordering or causality. The mediation estimates should therefore be interpreted as theory-informed indirect associations. Longitudinal, time-lagged, and experimental studies are needed to test whether changes in course experience precede changes in psychological need satisfaction, health belief, and subsequent exercise adherence behavior (Maxwell and Cole, 2007; Rohrer, 2018).

All focal variables were measured with self-report questionnaires. Although procedural controls, Harman’s test, and the single-factor CFA did not indicate a dominant common-method factor, these procedures cannot fully rule out same-source bias. Social desirability, recall error, and consistency motifs may still influence the estimates (Brenner and DeLamater, 2016; Motl et al., 2005). Future studies could combine questionnaires with attendance records, exercise logs, wearable-device data, fitness-app records, or teacher-rated engagement.

EA represented current self-reported tendency toward regular, persistent, and effortful exercise, not objectively verified lifelong behavior. The results should therefore not be generalized directly to exercise maintenance after graduation. Following students across semesters and into the post-university transition would provide stronger evidence about sustained behavior when course requirements are removed (Zhao et al., 2026).

The sample comprised undergraduates from universities in Sichuan Province who were currently taking or had recently completed a PE course. Generalizability to other regions, private universities, vocational colleges, graduate students, or students without recent PE experience is uncertain. Replication across regions and institution types, together with multi-group analysis by gender, grade, sport-organization participation, and baseline exercise frequency, would be informative.

The item-level hierarchical CFA supported the 16 first-order dimensions and four second-order constructs, but the primary structural model used dimension-level means. Its very favorable fit indices should therefore be understood as close reproduction of the dimension-level covariance matrix. The covariate-adjusted and sensitivity models showed stable focal paths, yet observed covariate adjustment cannot eliminate unmeasured confounding. Institution-, class-, and teacher-level cluster identifiers were not available in the analytical dataset, so multilevel SEM could not be conducted. Future research should retain these identifiers and examine contextual variation across courses, teachers, and universities.

The fsQCA findings depend on the selected conditions, calibration anchors, directional assumptions, and truth-table thresholds. Alternative anchors and frequency thresholds produced similar patterns, but the configurations remain set-theoretic associations within the present sample. Future studies could add peer support, family exercise culture, digital fitness engagement, sport-club involvement, campus policy, and objective facility access, and could use qualitative case evidence to enrich configuration interpretation (Greckhamer et al., 2018).

The configurational analysis focused on high EA. Non-high EA may involve asymmetric patterns rather than the simple absence of conditions associated with high EA. A dedicated analysis of non-high adherence could identify distinct combinations relevant to students reporting weak motivation, limited health belief, poor course experience, or restricted exercise opportunities.

## 6 Conclusion

This study examined exercise adherence tendency among undergraduates in Sichuan Province from chain-mediation and configurational perspectives. Integrated PE course experience was positively associated with exercise adherence tendency, and most of the total association was represented by indirect associations through exercise psychological need satisfaction and health belief. The sequential EPNS-HB pathway was the largest specific indirect component. fsQCA further identified six partially overlapping sufficient configurations, with HB present in all and EPNS in five. Together, the findings show that average structural associations and conjunctural condition patterns offer complementary accounts of exercise adherence tendency. The results concern current self-reported tendencies and should not be interpreted as evidence of causal or lifelong behavioral effects.

## Data Availability

The original contributions presented in the study are included in the article/Supplementary material, further inquiries can be directed to the corresponding author.

## Glossary

IPECE: integrated physical education course experience
EPNS: exercise psychological need satisfaction
HB: health belief
EA: exercise adherence tendency
TC: teaching characteristics
TCA: teacher characteristics and attitudes
CE: course experience
LA: learning atmosphere
FA: facilities
EX: examination
AUT: autonomy need satisfaction
COM: competence need satisfaction
REL: relatedness need satisfaction
HA: health awareness
PS: perceived susceptibility to health risks
DS: perceived severity of health risks
SE: self-efficacy
BH: behavioral habits
EC: effort commitment
EE: emotional experience
