# Supplementary material for: Integrated physical education course experience and exercise adherence tendency among undergraduates in Sichuan Province, China: chain mediation and configurational evidence from SEM and fsQCA
Source: Front Psychol. 2026 Aug 18;17:1902785. doi: 10.3389/fpsyg.2026.1902785 (PMC13529511; doi:10.3389/fpsyg.2026.1902785)
Supplement: Supplementary file 1 [file Table_1.DOCX]

**Supplementary Tables**

**Integrated Physical Education Course Experience and Exercise Adherence Tendency among Undergraduates in Sichuan Province, China: Chain Mediation and Configurational Evidence from SEM and fsQCA**

**Table S1. Fit indices for the item-level confirmatory factor analysis models.**

| **Model** | **χ²** | **df** | **χ²/df** | **CFI** | **TLI** | **SRMR** | **RMSEA** | **90% CI** | **AIC** | **BIC** |
| --- | --- | --- | --- | --- | --- | --- | --- | --- | --- | --- |
| Correlated 16 first-order factors | 2,445.374 | 2,090 | 1.170 | 0.997 | 0.997 | 0.013 | 0.007 | [0.006, 0.008] | 596,789.299 | 598,358.292 |
| Four second-order constructs over 16 first-order factors | 2,540.886 | 2,188 | 1.161 | 0.997 | 0.997 | 0.015 | 0.007 | [0.006, 0.008] | 596,688.811 | 597,657.174 |

Note. Both models converged without inadmissible estimates. The four-second-order-construct model did not fit significantly worse than the correlated 16-factor model, Δχ²(98) = 95.512, p = 0.552, and was retained as the more parsimonious hierarchical representation. CFI = comparative fit index; TLI = Tucker-Lewis index; SRMR = standardized root mean square residual; RMSEA = root mean square error of approximation; AIC = Akaike information criterion; BIC = Bayesian information criterion.

**Table S2. Standardized item loadings from the item-level hierarchical CFA.**

| **Construct** | **Dimension** | **Item** | **Loading** | **SE** | **z** | **p** | **95% CI** |
| --- | --- | --- | --- | --- | --- | --- | --- |
| IPECE | TC | X_TC_1 | 0.808 | 0.009 | 92.102 | < 0.001 | [0.790, 0.825] |
| IPECE | TC | X_TC_2 | 0.729 | 0.010 | 71.269 | < 0.001 | [0.708, 0.749] |
| IPECE | TC | X_TC_3 | 0.764 | 0.010 | 80.066 | < 0.001 | [0.745, 0.783] |
| IPECE | TCA | X_TCA_1 | 0.730 | 0.009 | 78.282 | < 0.001 | [0.712, 0.749] |
| IPECE | TCA | X_TCA_2 | 0.699 | 0.010 | 69.456 | < 0.001 | [0.679, 0.718] |
| IPECE | TCA | X_TCA_3 | 0.731 | 0.009 | 78.542 | < 0.001 | [0.713, 0.749] |
| IPECE | TCA | X_TCA_4 | 0.757 | 0.009 | 87.071 | < 0.001 | [0.740, 0.774] |
| IPECE | TCA | X_TCA_5 | 0.715 | 0.010 | 73.716 | < 0.001 | [0.696, 0.734] |
| IPECE | TCA | X_TCA_6 | 0.737 | 0.009 | 80.391 | < 0.001 | [0.719, 0.755] |
| IPECE | CE | X_CE_1 | 0.755 | 0.009 | 80.456 | < 0.001 | [0.736, 0.773] |
| IPECE | CE | X_CE_2 | 0.676 | 0.011 | 61.120 | < 0.001 | [0.654, 0.698] |
| IPECE | CE | X_CE_3 | 0.768 | 0.009 | 84.289 | < 0.001 | [0.750, 0.786] |
| IPECE | CE | X_CE_4 | 0.759 | 0.009 | 81.690 | < 0.001 | [0.741, 0.777] |
| IPECE | LA | X_LA_1 | 0.730 | 0.010 | 71.093 | < 0.001 | [0.710, 0.750] |
| IPECE | LA | X_LA_2 | 0.647 | 0.012 | 54.216 | < 0.001 | [0.624, 0.671] |
| IPECE | LA | X_LA_3 | 0.727 | 0.010 | 70.377 | < 0.001 | [0.707, 0.747] |
| IPECE | LA | X_LA_4 | 0.736 | 0.010 | 72.431 | < 0.001 | [0.716, 0.756] |
| IPECE | FA | X_FA_1 | 0.755 | 0.009 | 85.521 | < 0.001 | [0.737, 0.772] |
| IPECE | FA | X_FA_2 | 0.736 | 0.009 | 79.343 | < 0.001 | [0.717, 0.754] |
| IPECE | FA | X_FA_3 | 0.755 | 0.009 | 85.763 | < 0.001 | [0.738, 0.773] |
| IPECE | FA | X_FA_4 | 0.764 | 0.009 | 88.938 | < 0.001 | [0.748, 0.781] |
| IPECE | FA | X_FA_5 | 0.756 | 0.009 | 85.892 | < 0.001 | [0.739, 0.773] |
| IPECE | EX | X_EX_1 | 0.719 | 0.010 | 70.647 | < 0.001 | [0.699, 0.739] |
| IPECE | EX | X_EX_2 | 0.741 | 0.010 | 76.539 | < 0.001 | [0.722, 0.760] |
| IPECE | EX | X_EX_3 | 0.743 | 0.010 | 76.854 | < 0.001 | [0.724, 0.762] |
| IPECE | EX | X_EX_4 | 0.761 | 0.009 | 82.116 | < 0.001 | [0.743, 0.779] |
| EPNS | AUT | M1_AUT_1 | 0.758 | 0.009 | 80.654 | < 0.001 | [0.740, 0.777] |
| EPNS | AUT | M1_AUT_2 | 0.754 | 0.010 | 79.342 | < 0.001 | [0.735, 0.772] |
| EPNS | AUT | M1_AUT_3 | 0.751 | 0.010 | 78.599 | < 0.001 | [0.732, 0.770] |
| EPNS | AUT | M1_AUT_4 | 0.710 | 0.010 | 68.220 | < 0.001 | [0.690, 0.731] |
| EPNS | COM | M1_COM_1 | 0.744 | 0.010 | 76.952 | < 0.001 | [0.725, 0.763] |
| EPNS | COM | M1_COM_2 | 0.741 | 0.010 | 76.177 | < 0.001 | [0.722, 0.760] |
| EPNS | COM | M1_COM_3 | 0.748 | 0.010 | 78.069 | < 0.001 | [0.729, 0.767] |
| EPNS | COM | M1_COM_4 | 0.738 | 0.010 | 75.428 | < 0.001 | [0.719, 0.757] |
| EPNS | REL | M1_REL_1 | 0.750 | 0.010 | 78.737 | < 0.001 | [0.732, 0.769] |
| EPNS | REL | M1_REL_2 | 0.746 | 0.010 | 77.501 | < 0.001 | [0.727, 0.765] |
| EPNS | REL | M1_REL_3 | 0.754 | 0.009 | 79.889 | < 0.001 | [0.736, 0.773] |
| EPNS | REL | M1_REL_4 | 0.724 | 0.010 | 71.780 | < 0.001 | [0.704, 0.744] |
| HB | HA | M2_HA_1 | 0.790 | 0.007 | 105.511 | < 0.001 | [0.775, 0.804] |
| HB | HA | M2_HA_2 | 0.786 | 0.008 | 103.650 | < 0.001 | [0.771, 0.801] |
| HB | HA | M2_HA_3 | 0.711 | 0.009 | 75.349 | < 0.001 | [0.692, 0.729] |
| HB | HA | M2_HA_4 | 0.798 | 0.007 | 109.695 | < 0.001 | [0.784, 0.812] |
| HB | HA | M2_HA_5 | 0.799 | 0.007 | 110.492 | < 0.001 | [0.785, 0.814] |
| HB | HA | M2_HA_6 | 0.787 | 0.008 | 104.019 | < 0.001 | [0.772, 0.801] |
| HB | PS | M2_PS_1 | 0.836 | 0.007 | 113.866 | < 0.001 | [0.822, 0.850] |
| HB | PS | M2_PS_2 | 0.848 | 0.007 | 119.022 | < 0.001 | [0.834, 0.862] |
| HB | PS | M2_PS_3 | 0.760 | 0.009 | 85.396 | < 0.001 | [0.742, 0.777] |
| HB | DS | M2_DS_1 | 0.766 | 0.010 | 78.571 | < 0.001 | [0.746, 0.785] |
| HB | DS | M2_DS_2 | 0.763 | 0.010 | 77.848 | < 0.001 | [0.743, 0.782] |
| HB | DS | M2_DS_3 | 0.752 | 0.010 | 75.298 | < 0.001 | [0.732, 0.772] |
| HB | SE | M2_SE_1 | 0.583 | 0.013 | 44.848 | < 0.001 | [0.557, 0.608] |
| HB | SE | M2_SE_2 | 0.747 | 0.010 | 77.225 | < 0.001 | [0.728, 0.766] |
| HB | SE | M2_SE_3 | 0.828 | 0.008 | 101.704 | < 0.001 | [0.812, 0.844] |
| HB | SE | M2_SE_4 | 0.723 | 0.010 | 71.150 | < 0.001 | [0.703, 0.743] |
| EA | BH | Y_BH_1 | 0.875 | 0.006 | 156.304 | < 0.001 | [0.864, 0.886] |
| EA | BH | Y_BH_2 | 0.849 | 0.006 | 137.267 | < 0.001 | [0.837, 0.861] |
| EA | BH | Y_BH_3 | 0.762 | 0.008 | 91.774 | < 0.001 | [0.746, 0.778] |
| EA | BH | Y_BH_4 | 0.768 | 0.008 | 94.211 | < 0.001 | [0.752, 0.784] |
| EA | EC | Y_EC_1 | 0.738 | 0.009 | 83.631 | < 0.001 | [0.721, 0.756] |
| EA | EC | Y_EC_2 | 0.742 | 0.009 | 84.940 | < 0.001 | [0.725, 0.759] |
| EA | EC | Y_EC_3 | 0.858 | 0.006 | 146.505 | < 0.001 | [0.846, 0.869] |
| EA | EC | Y_EC_4 | 0.755 | 0.008 | 89.567 | < 0.001 | [0.738, 0.771] |
| EA | EC | Y_EC_5 | 0.697 | 0.010 | 71.162 | < 0.001 | [0.678, 0.717] |
| EA | EC | Y_EC_6 | 0.776 | 0.008 | 98.324 | < 0.001 | [0.761, 0.792] |
| EA | EE | Y_EE_1 | 0.764 | 0.009 | 83.187 | < 0.001 | [0.746, 0.782] |
| EA | EE | Y_EE_2 | 0.771 | 0.009 | 85.111 | < 0.001 | [0.753, 0.788] |
| EA | EE | Y_EE_3 | 0.673 | 0.011 | 60.421 | < 0.001 | [0.651, 0.695] |
| EA | EE | Y_EE_4 | 0.758 | 0.009 | 81.247 | < 0.001 | [0.739, 0.776] |

Note. All loadings were estimated in the four-second-order-construct hierarchical CFA. IPECE = integrated physical education course experience; EPNS = exercise psychological need satisfaction; HB = health belief; EA = exercise adherence tendency; TC = teaching characteristics; TCA = teacher characteristics and attitudes; CE = course experience; LA = learning atmosphere; FA = facilities; EX = examination; AUT = autonomy need satisfaction; COM = competence need satisfaction; REL = relatedness need satisfaction; HA = health awareness; PS = perceived susceptibility; DS = perceived severity; SE = self-efficacy; BH = behavioral habits; EC = effort commitment; EE = emotional experience.

**Table S3. Reliability and convergent validity of the 16 first-order dimensions.**

| **Construct** | **Dimension** | **Dimension name** | **Items** | **Loading range** | **Cronbach’s α** | **CR** | **AVE** |
| --- | --- | --- | --- | --- | --- | --- | --- |
| IPECE | TC | Teaching characteristics | 3 | .729-.808 | .810 | .811 | .589 |
| IPECE | TCA | Teacher characteristics and attitudes | 6 | .699-.757 | .869 | .871 | .531 |
| IPECE | CE | Course experience | 4 | .676-.768 | .829 | .829 | .548 |
| IPECE | LA | Learning atmosphere | 4 | .647-.736 | .797 | .803 | .505 |
| IPECE | FA | Facilities | 5 | .736-.764 | .868 | .868 | .567 |
| IPECE | EX | Examination | 4 | .719-.761 | .828 | .830 | .549 |
| EPNS | AUT | Autonomy need satisfaction | 4 | .710-.758 | .832 | .832 | .553 |
| EPNS | COM | Competence need satisfaction | 4 | .738-.748 | .831 | .831 | .552 |
| EPNS | REL | Relatedness need satisfaction | 4 | .724-.754 | .832 | .832 | .553 |
| HB | HA | Health awareness | 6 | .711-.799 | .902 | .902 | .607 |
| HB | PS | Perceived susceptibility | 3 | .760-.848 | .853 | .856 | .665 |
| HB | DS | Perceived severity | 3 | .752-.766 | .804 | .804 | .578 |
| HB | SE | Self-efficacy | 4 | .583-.828 | .807 | .814 | .527 |
| EA | BH | Behavioral habits | 4 | .762-.875 | .886 | .888 | .665 |
| EA | EC | Effort commitment | 6 | .697-.858 | .890 | .893 | .582 |
| EA | EE | Emotional experience | 4 | .673-.771 | .828 | .830 | .551 |

Note. All standardized item loadings were significant at p < 0.001. CR = composite reliability; AVE = average variance extracted. Abbreviations are defined in Table S2.

**Table S4. Standardized first-order dimension loadings on the four second-order constructs in the item-level hierarchical CFA.**

| **Construct** | **Dimension** | **Loading** | **SE** | **z** | **p** | **95% CI** | **CR** | **AVE** |
| --- | --- | --- | --- | --- | --- | --- | --- | --- |
| IPECE | TC | 0.760 | 0.011 | 68.320 | < 0.001 | [0.739, 0.782] | .889 | .571 |
| IPECE | TCA | 0.759 | 0.010 | 73.626 | < 0.001 | [0.739, 0.779] |  |  |
| IPECE | CE | 0.748 | 0.011 | 67.344 | < 0.001 | [0.726, 0.770] |  |  |
| IPECE | LA | 0.760 | 0.011 | 67.755 | < 0.001 | [0.738, 0.782] |  |  |
| IPECE | FA | 0.756 | 0.010 | 72.434 | < 0.001 | [0.736, 0.777] |  |  |
| IPECE | EX | 0.750 | 0.011 | 67.561 | < 0.001 | [0.728, 0.772] |  |  |
| EPNS | AUT | 0.726 | 0.013 | 56.224 | < 0.001 | [0.701, 0.751] | .797 | .568 |
| EPNS | COM | 0.772 | 0.012 | 63.689 | < 0.001 | [0.748, 0.795] |  |  |
| EPNS | REL | 0.762 | 0.012 | 62.133 | < 0.001 | [0.738, 0.786] |  |  |
| HB | HA | 0.771 | 0.010 | 74.584 | < 0.001 | [0.751, 0.791] | .834 | .557 |
| HB | PS | 0.746 | 0.011 | 65.519 | < 0.001 | [0.724, 0.768] |  |  |
| HB | DS | 0.762 | 0.012 | 64.217 | < 0.001 | [0.739, 0.785] |  |  |
| HB | SE | 0.705 | 0.013 | 55.922 | < 0.001 | [0.680, 0.730] |  |  |
| EA | BH | 0.771 | 0.011 | 69.449 | < 0.001 | [0.749, 0.792] | .813 | .591 |
| EA | EC | 0.773 | 0.011 | 70.134 | < 0.001 | [0.751, 0.794] |  |  |
| EA | EE | 0.763 | 0.012 | 63.782 | < 0.001 | [0.740, 0.787] |  |  |

Note. These estimates were obtained from the 68-item hierarchical CFA and are analytically distinct from the dimension-level loading ranges reported in Table 2 of the main manuscript. CR and AVE are reported once for each second-order construct. CR = composite reliability; AVE = average variance extracted. Abbreviations are defined in Table S2.

**Table S5. Robustness and sensitivity of the focal SEM associations and explained variance.**

**Panel A. Standardized focal structural associations**

| **Path** | **Primary model β** | **Covariate-adjusted β** | **Sensitivity model β** |
| --- | --- | --- | --- |
| IPECE → EA | .131 | .127 | .127 |
| IPECE → EPNS | .759 | .760 | .760 |
| EPNS → EA | .145 | .150 | .150 |
| IPECE → HB | .327 | .327 | .328 |
| HB → EA | .568 | .568 | .567 |
| EPNS → HB | .486 | .485 | .484 |

**Panel B. R² for endogenous constructs**

| **Endogenous construct** | **Primary model** | **Covariate-adjusted model** | **Sensitivity model** |
| --- | --- | --- | --- |
| EPNS | .576 | .581 | .582 |
| HB | .584 | .587 | .587 |
| EA | .614 | .618 | .618 |

Note. All six focal associations were significant at p < 0.001 in all three models. Age was entered as a continuous covariate. Gender, grade, academic discipline, university type, PE-course status, and sport-organization participation were represented by k−1 dummy variables. The reference categories were male, first-year students, humanities and social sciences, comprehensive universities, current PE-course participation, and no sport-organization participation. All covariates were entered simultaneously as predictors of EPNS, HB, and EA, and covariances among the covariates were freely estimated. The sensitivity model additionally included three dummy variables for weekly exercise frequency, with 0–1 time per week as the reference category. Model fit was χ²(98) = 92.763, CFI = 1.000, TLI = 1.000, SRMR = 0.009, RMSEA = 0.000 for the primary model; χ²(332) = 327.482, CFI = 1.000, TLI = 1.000, SRMR = 0.011, RMSEA = 0.000 for the covariate-adjusted model; and χ²(371) = 366.643, CFI = 1.000, TLI = 1.000, SRMR = 0.011, RMSEA = 0.000 for the sensitivity model. IPECE = integrated physical education course experience; EPNS = exercise psychological need satisfaction; HB = health belief; EA = exercise adherence tendency.
